# Depression and medication-adherence in patients with hypertension attending a tertiary health facility in South-West Nigeria

**DOI:** 10.11604/pamj.2019.33.27.12941

**Published:** 2019-05-15

**Authors:** Hannah Iyabo Okunrinboye, Alexander Ndubusi Otakpor, Olayinka Stephen Ilesanmi

**Affiliations:** 1 Department of Psychiatry, Federal Medical Centre, Owo, Ondo State, Nigeria; 2 Department of Mental Health, University of Benin Teaching Hospital, Benin City, Edo state, Nigeria; 3 Department of Community Medicine, University of Ibadan, Ibadan, Oyo State, Nigeria

**Keywords:** Depression, hypertension, medication adherence

## Abstract

**Introduction:**

In Nigeria, approximately 4.33 million adults suffer from hypertension and about a third of them do not adhere to prescribed medications. Depression has been reported to significantly predict poor medication adherence. The relationship between medication non-adherence and co-morbid depressive disorder in patients with hypertension has not been adequately explored in this environment. The study aimed to determine the prevalence of depression in patients with hypertension. The association between socio-demographic characteristics and presence of co-morbidity on medication adherence was also determined.

**Methods:**

A cross-sectional descriptive research design was adopted for the study. A socio-demographic questionnaire, the modified Morisky Medication Adherence Scale (MMAS), the Hamilton Rating Scale for Depression (HAM-D) and the Mini International Neuropsychiatric Interview (MINI), were administered to four hundred patients with hypertension attending medical out-patient clinic between August and September 2012.

**Results:**

About 43% (168) were aged 61 to 64 years the majority being females, with a female to male ratio of 1.63:1. The prevalence of comorbid depression was 22.8%, made up of mild (21.8%) and moderate (1.0%) depressive episodes only. Depression was commoner among females than males in a ratio of 3:1. A majority of the participants (96.8%) had high medication adherence; 2.8% and 0.4% had moderate and low adherence respectively. Depression was more among patients with good medication adherence.

**Conclusion:**

The occurrence of mild depressive disorder among hypertensives did not affect the level of medication adherence. Review of Antihypertensive drugs should also be done often to ensure patients are not likely to have depressive illness as a side effect of drugs used.

## Introduction

Depression is a very common mental health problem worldwide. The World Health Organization (WHO) estimates that 121 million people currently suffer from depression and that depression will become the second most common cause of disability after heart disease by 2020 [1]. Depression often co-occurs with chronic physical illnesses such as hypertension, diabetes mellitus and epilepsy [2-4]. These chronic physical illnesses sometimes lead to a decrease in the patient's quality of life and thus increase the risk of developing depression. Compared with healthy people, cardiac patients appear to be at greater risk of developing depression many years after the initial medical diagnosis is made [5]. The physiological mechanisms underlying the relationship between depression and hypertension are complex, intricately interwoven and appear to be largely mediated by neurotransmitter effects involving the sympathetic nervous system [6]. For instance, some drugs used in the treatment of hypertension such as methyldopa and reserpine are known to precipitate depression, while some of the drugs used in the treatment of depression like bupropion, venlafaxine and phenelzine have been reported to cause hypertension [7, 8].

Medication adherence refers to patient's use of medications in strict compliance with the prescription [9]. Poor medication adherence is a significant risk factor for poor treatment outcome in patients with hypertensive disease particularly with the associated high rate of preventable deaths. Uncontrolled blood pressure often from non-adherence to medication use, is associated with such untoward sequelae as sudden deaths, stroke and renal failure. In a Nigerian study involving patients attending a secondary health facility in Oyo State, only about a third (33.6%) of the cases were reported to have controlled blood pressure (CBP) and this was partly blamed on poor adherence to antihypertensive medications. Depression has been reported to significantly predict poor medication adherence [10-12]. About a third of persons with physical ailment in Nigeria, on the average, have a co-existing mental disorder [13]. This modifiable risk factor, when identified, can be well managed by physicians thereby improving adherence to medication and avoid the attendant dangerous health consequences of non-compliance. Despite the mounting evidence regarding the association between poor medication adherence, depression, and uncontrolled blood pressure, assessment of patients' adherence to prescribed medications, screening for depression and use of individualised interventions to improve adherence are rarely carried out in routine clinical practice [9]. The study aimed to determine the prevalence of depression in patients with hypertension. The association between socio-demographic characteristics and presence of comorbidity on medication adherence was also determined. It is hoped that the findings will serve as baseline data for working out appropriate intervention protocol for the clinic services, for future medical education and health campaign programs.

## Methods

**Study setting:** this study was carried out at the Medical Out-patient Clinics (MOPC) of the Federal Medical Centre (FMC) Owo, Ondo State, Nigeria; it is the only tertiary hospital in the state. Owo is the headquarters of Owo Local Government Area (LGA), one of the 18 LGAs in Ondo State. Owo is situated in southwestern Nigeria, at the southern edge of the Yoruba hills and at the intersection of roads from Akure, Kabba and Benin-City. It is predominantly inhabited by the Yoruba ethnic group. Yoruba and English are the main languages of communication. The Centre has five consultant physicians and ten medical officers running its Medical Outpatient Clinic with a consultant supervising a clinic every weekday; averages of 20-30 patients with hypertension visit the clinic each weekday. The facility is opened from 8am to 4pm on week days and closes for weekends.

**Study design:** the study is descriptive and cross-sectional in design.

**Study population:** the study population was made up of patients attending the the Federal Medical Centre (FMC) Owo during the study period; from amongst those between the ages of 18 and 64 years who were diagnosed by a consultant physician at the Centre as suffering from hypertension and have been on anti-hypertensive medication for at least 6 months, spoke Yoruba or English language fluently and gave consent to participate in the study was the sample recruited. Severely ill patients and those who did not give consent were excluded.

**Ethical consideration:** approval to carry out the study was obtained from the Ethics committee of the Federal Medical Centre Owo, Ondo State.

**Sample size calculation:** in an earlier study conducted in Nigeria, the prevalence of medication non-adherence among hypertensive patients was found to be 34.2% [14]. Using appropriate statistical formulae, with 10% non-response rate and probability of making type 1 error of 5%.

$$N=\frac{z^{2}pq}{d^{2}}$$

Where N= minimum sample size Z = 1.96 (95% confidence interval) p = prevalence of the variable in question=34.2% [14]. q = 1-p d^2^ = 0.05^2^ (Value of Z = 1.96, p = 0.342, q = 0.658, d = 0.05) Therefore

$$N=\frac{{\left(1.92\right)}^{2}\times \;0.342\times 0.658}{0.052}=345.80$$

A sample size of 400 participants was recruited to enhance the reliability of the results [15].

**Sampling method:** systematic random sampling method was used to select 12 patients daily for a period of 7 weeks (35 clinic days). The daily sampling frame was made up of 20-30 eligible patients. The sampling interval was 30/12, that is 2. From the first three patients seen daily one was randomly selected, then every third patient was selected until a total number of 12 patients were seen.

**Measures and assessments:** the instruments used in this study were designed to elicit the socio-demographic and clinical characteristics of the patients, determine the presence and severity of depression and medication adherence. The questionnaires were administered in English or Yoruba.

**The Mini International Neuro-psychiatric Interview (MINI):** clinical diagnosis of depression was done using the Mini International Neuro-psychiatric Interview (MINI) Schedule which is a short structured diagnostic interview. The MINI. is divided into modules identified by letters corresponding to the diagnostic categories. Depressive module of MINI is grouped as A-module and has six sections A1 to A6. When the M.I.N.I. screen is implemented properly, it increases the likelihood of identifying someone who truly has mental illness. The MINI. is not to be self-administered, neither does it require any formal training before it can be used. The MINI has been used in medical out-patient populations in Nigeria [2] and student populations [16]; it has a sensitivity of 0.70 and specificity of 0.85 for major depressive disorder.

**Hamilton rating scale for depression:** severity of the depression identified by MINI was assessed using the Hamilton Rating Scale for Depression (HAM-D); it is used to detect the presence and rate the severity of depressive signs and symptoms in study participants. It consists of 21 items with a possible maximum score of 66 and a minimum score of 1. HAMD ratings are made on the basis of the clinical interview, plus any additional available information such as nursing or family member report. Higher scores indicate severe depression as in the HAM-D is a well validated instrument and has an excellent research base. Its sensitivity is 86.4% and specificity is 92.2%. HAM-D has been used in medical out-patient settings in Nigeria [3, 4].

**Morisky medication adherence scale:** it is an 8-item (MMAS-8) questionnaire that was used to ascertain medication-adherence by the patients. It is a self-reported measure of medication taking which was developed from a previously validated four-item scale and supplemented with additional items addressing the circumstances surrounding adherence behaviour. Each item on the scale measures a specific medication-taking behaviour and not a determinant of adherence behaviour. It has a higher sensitivity (0.93), reliability (0.83) and more satisfactory specificity (0.53) than the 4-item version, MMAS-4. The scores ranged from zero to eight, with scores of 0 to 5, 6 to 7, and 8 reflecting low, medium and high adherence respectively. It has been used in Nigeria by Adewuya and colleagues among patients with psychiatric illness in hospital based studies in the Southwestern part of Nigeria [17]. For the purpose of this research, the researcher translated the questionnaires to Yoruba language (target language) using the method of back-translation. Back-translation method was used by the World Health Organisation (WHO) in preparing the Present State Examination (PSE) Schedule in seven different languages for the International Study of Schizophrenia (ISoS) [18].

**Procedure:** the case-notes of patients attending the Medical out Patient Clinic Department (MOPCD) of the Centre during the study period were examined each clinic day to select out possible cases to be recruited. Four hundred and fifteen patients were approached, five declined to participate; and of the 410 that took part initially, ten of them withdrew during the interview for varied reasons. Thus 400 participants completed the study. Systematic random sampling method was used to select 12 patients daily for a period of seven weeks, five clinic days per week. The daily sampling frame was made up of 20-30 eligible patients. The sampling interval was 30/12, that is 2. From the first three patients seen daily one was randomly selected, then every third patient was selected until a total number of 12 patients were seen daily. The nature and purpose of the study was explained to them and their care givers (when present); they were assured of confidentiality and following consent to participate, the researcher administered the socio-demographic questionnaire, the depression module of the Mini International Neuropsychiatric Interview (MINI), Hamilton Rating Scale for Depression (HAM-D) and the 8-item MoriskyMedication Adherence scale in that order. It took the researcher between 30 - 45 minutes to administer the instruments to each patient. Comorbidity is the occurrence of two disorders or illnesses in the same person simultaneously with a resultant effect on the prognosis. Good adherence are patients who take the prescribed number of pills each day and within a prescribed period, while those who did not were said to have had poor adherence.

**Data analysis:** the data were analysed using the Statistical Package for Social Sciences software (SPSS) Version 17 and the results were summarised using descriptive statistics and presented in tables. Associations between categorical and continuous variables were tested using the chi-square and student t-test respectively. Socio-demographic and illness variables were entered into a logistic regression model to determine predictors of poor medication-adherence. Level of significance was 0.05.

## Results

Table 1 shows the socio-demographic characteristics of the respondents. The mean age of respondents was 59.6 +/- 8.0 years. The age range of the respondents was 23-64 years with a median age of 60 years. In all, 229 (57.3%) were in the age group of ≥60 years, while 248 (62.0%) of them were females. Also 342 (85.5%) of the respondents were Christians. Married respondents were 277 (69.3%), while 243 (60.7%) had co-morbid conditions. Considering educational status, the respondents without formal education were 72 (18.0%) while those with tertiary education were 126 (31.5%). Also, 207 (51.8%) of the respondents were employed. With respect to the income and amount spent on treatment per month, 212 (53.0%) of the respondents earned ≥ N20,000 monthly, while 311 (77.8%) spent ≤ N5000 monthly. Concerning depression distribution among the participants 87 (21.8%) had mild depressive episode, 4 (1.0%) had moderate depressive episode, while 309(77.2%) did not have depression. The majority, 387 (96.8%), had high adherence while 13 (3.2%) had low medication adherence. Table 2 shows the relationship between socio-demographic factors of the participants with and without diagnosis of depression. Depression was present in 59 (23.8%) of female respondents compared to 22 (14.5%) of male respondents (p=0.024). Twenty-three (31.9%) of the respondents without formal education had depression compared to 14 (11.1%) of those with tertiary education (p=0.002). Of non-married respondents 37 (30.1%) were more likely to have depression compared to 44 (15.9%) of married respondents. This is statistically significant with a p value of 0.001. Depression was commoner among 52 (27.7%) of respondents that earned < N20,000 per month compared to 29 (13.7%) of those who earned ≥ N20,000 per month. Even though respondents aged 60 years and above, those practicing other religion apart from Christianity, the unemployed, those with co-morbidity and those who earned ≤ N5000 per month were more likely to have depression, these were not statistically significant.

**Table 1:** Socio demographic characteristics of the study participants

Variables	Frequency	%
**Age in years**		
<60	171	42.8
≥60	229	57.2
**Gender**		
Male	152	38.0
Female	248	62.0
**Religion**		
Christianity	342	85.5
Others	58	14.5
**Marital Status**		
Married	277	69.3
Not married (separated, divorced, widow)	123	30.7
**Educational Status**		
No formal education	72	18.0
Primary	108	27.0
Secondary	94	23.5
Tertiary	126	31.5
**Employment status**		
Employed	207	51.8
Un Employed	193	48.2
**Monthly Income**		
<₦20,000	188	47.0
≥₦20,000	212	53.0
**Amount spent on Treatment per month**		
≤₦5000	311	77.8
>₦5000	89	22.2
**Co morbid condition**		
None	157	39.3
Present	243	60.7

**Table 2:** Comparison of socio-demographic factors of the study participants with and without diagnosis of depression

Characteristics	Depression absent N (%)	Depression Present N (%)	X^2^	p-value
**Age in years**				0.245
˂60	141(82.5%)	30(17.5%)	1.352	
≥60	178(77.7%)	51(22.3%)		
**Gender**				0.024
Female	189(76.2%)	59(23.8%)	0.024	
Male	130(85.5%)	22(14.5%)		
**Religion**				0.792
Christianity	272(79.5%)	70(20.5%)	0.069	
Others	47(81.0%)	11(19.0%)		
**Marital Status**				0.001
Married	233(84.1%)	44(15.9%)	10.630	
Not Married	86(69.9%)	37(30.1%)		
**Employment status**				0.633
Employed	167(80.7%)	40(19.3%)	0.228	
Unemployed	152(78.8%)	41(21.2%)		
Co-morbidity*				0.648
None	127(80.9%)	30(19.1%)	0.209	
Present	192(79.0%)	51(21.0%)		
**Educational Status**				0.002
No formal education	49(68.1%)	23(31.9%)	14.395	
Primary	81(75.0%)	27(25.0%)		
Secondary	77(81.9%)	17(18.1%)		
Tertiary	112(88.9%)	14(11.1%)		
**Income per Month**				0.001
˂₦20,000	136(72.3%)	52(27.7%)	12.059	
≥₦20,000	183(86.3%)	29(13.7%)		
**Amount spent on Treatment**				0.366
≤₦5000	245(78.8%)	66(21.2%)	0.817	
˃₦5000	74(83.1%)	15(16.9%)		

* Co-morbidity is the occurrence of two disorders or illnesses in the same person simultaneously with a resultant effect on the prognosis.

Table 3 shows the association between socio-demographic characteristics of the participants and medication adherence. In all, 10 (6.4%) respondents that do not have co-morbid condition were likely to have poor adherence to medication compared to 4 (1.6%) with co-morbid condition (p=0.012). Also 355(97.0%) of respondents with effective perception of medication have good medication adherence compared to 31 (91.2%) of respondents who do not perceive their medication as effective (p=0.077). Good medication adherence was higher (96.9%) among those aged 60years and above compared to those less than 60years (95.9%) with a p-value of 0.577. Employment status, educational status and income per month did not really affect medication adherence among the respondents. The determinant of depression among study participant is shown in Table 4. Female had 1.85 unadjusted odds ratio of being depressed compared to male. Comparison of medication adherence and depression showed that among the participants with good medication adherence 79 (20.5%) had depression, while 2 (14.3%) of participants with poor medication adherence had depression. Unmarried respondents compared to married, those with primary education and below compared to secondary and above and those who earned less < N20,000 compared to ≥ N20,000 had two times likelihood of having depression.

**Table 3:** Comparison of socio-demographic characteristics and the level of medication adherence of participants

Variables	Medication adherence		X^2^	P-Value
	Good	Poor		
**Age in years**			0.312	0.577
˂60	164 (95.9%)	7 (4.1%)		
≥60	222 (96.9%)	7 (3.1%)		
**Religion**			2.460 0.112	0.117 2.525
Christianity	328 (95.9%)	14 (4.1%)		
Others	58 (100%)	0		
**Marital status**				
Married	270 (97.5%)	7 (2.5%)		
Not married	116 (94.3%)	7 (5.7%)		
**Employment Status**			0.018	0.894
Employed	200 (96.6%)	7 (3.4%)		
Unemployed	186 (96.4%)	(7 3.6%)		
**Co- morbidity**			6.300	0.012
None	147 (93.6%)	10 (6.4%)		
Present	239 (98.4%)	4 (1.6%)		
**Educational Status**			0.147	0.702
Primary and	173 (96.1%)	7 (3.9%)		
No formal education				
Secondary and Tertiary	213 (96.8%)	7 (3.2%)		
**Income**			0.052	0.819
˂ ₦ 20,000	181 (96.3%)	7 (3.7%)		
≥ ₦ 20,000	205 (96.7%)	7 (3.3%)		
**Amount spent on Treatment**			0.532	0.466
≤ ₦ 5000	299 (96.1%)	12 (3.9%)		
˃ ₦ 5000	87 (97.8%)	2 (2.2%)		
**Perception of Medication**			3.118	0.077
Effective	355 (97.0%)	11 (3.0%)		
Not effective	31 (91.2%)	3 (8.8%)		

**Table 4:** Determinants of depression among study participants

	Unadjusted analysis				Adjusted analysis			
Characteristics	Unadjusted Odds ratio	*CI of OR		p-value	Adjusted Odds ratio	CI of Adjusted OR		p-value
		Lower	Upper			Lower	Upper	
**Gender**								
Male	1				1			
Female	1.85	1.08	3.16	0.026	1.23	0.68	2.22	0.502
**Marital Status**								
Not Married	2.28	1.38	3.77	0.001	1.69	0.97	2.94	0.063
Married	1				1			
**Educational Status**								
Primary and below	2.35	1.42	3.87	0.001	1.52	0.83	2.79	0.174
Secondary and above	1				1			
**Income per month**								
**<**₦ 20,000	2.41	1.46	4.0	0.001	1.63	0.88	3.01	0.117
≥ ₦ 20,000	1				1			

* CI: Confidence interval

## Discussion

A prevalence rate of 22.8% for co-morbid depression in patients with hypertensive disease in this study is similar to the reported rate of 22% among outpatients with stable coronary heart disease in the United States, but would seem much higher than the 6.2% recently reported in patients with similar condition attending a cardiology clinic in Lagos, Nigeria [19, 20]. The Lagos study was noted to have excluded participants older than 60 years, an age group known to be vulnerable to developing depressive disorders for myriad psycho-social reasons; and the subgroup of patients who had developed complications from hypertension [20]. In this study, most of their identified cases had mild depressive disorder. The finding that depression was more prevalent among females compared to males with a female to male ratio of 3:1 is in keeping with the reports of earlier studies [21-23]. More prevalence of depression among females could be due to the fact that females use a high degree of ruminative coping and a low degree of distractive and problem-focused coping [23]. In addition, because females were nearly twice as likely to be included in the sample could have confounded the gender prevalence rate differentials observed. The non-married were found to be more amongst the cases with co-morbid depressive disorder than the married. Other researchers have found that participants with major depressive disorder have poor social support [21]. It is therefore understandable that married females tend to have good social support which could reduce their risk of developing depression. Religion, employment status and presence of co-morbidity, were not significantly associated with a diagnosis of depression. However, a population-based study in the United States reported that unemployment predicted greater odds of major depressive episode (MDE) among African American men, while there was an inverse relationship between level of education and 12-month MDE [24].

Also in this study, participants with depression were older, although this difference was not statistically significant. This is similar to a previous study report that depression occurred more in old age, reaches its lowest level in the middle age at about 45 years, and has a tendency to fall in early adulthood and rise in late life reflecting life-cycle gains and losses in marriage, employment and economic well-being [25]. Duration of formal education was significantly associated with depression in that participants with lower formal education had more co-morbid depression than those with higher formal educational attainment. This is because individuals with lower formal education tend to be more susceptible to the deleterious effects of negative cognitive and affective states [22]. More participants with lower income or allowance per month had depression compared to those who earned higher income; this is in keeping with Akhtar-Danesh and Landeen study finding which reported an inverse relationship between the level of income and the prevalence of depression [26]. Paradoxically, a higher proportion of those with co-morbidity had good medication adherence. Sometimes patients with co-morbidity take other medicines in addition to their hypertensive medications daily and this could have helped to improve their compliance. Also the good social support that the patients had could have been responsible for good medication adherence. However, some studies have shown that the presence of co-morbidity did not affect medication adherence [14, 27].

As shown in this study there was no significant relationship between age, marital status, educational status, monthly income, cost of treatment per month, and medication adherence. Previous studies had reported that old age and being married were independent predictors of medication adherence [28, 29]. This could be because older people that are married tend to have good social support which is associated with good medication adherence among patients with hypertension. Other studies found that having a strong social support system was associated with high medication adherence among patients with hypertension [30, 31]. The high cost of antihypertensive medications also contributes to non-adherence in developing countries where health care insurance facilities seldom exist. In a study conducted in Nigeria among patients with hypertension on treatment, 34.2% of them were non-adherent to their medication and lack of finance accounted for 23.8% of such cases [14]. In Ghana, 93% of hypertensive patients were non-adherent with medication regimens and 96% of such patients cited unaffordable drug prices as the main reason [32]. Even though in this study there was no significant relationship between age and medication adherence, a cross-sectional study of out-patients at a Veterans Health Administration site showed that concomitant use of more than one antihypertensive medication increases with patient's age and may have an impact on the patient's willingness or ability to comply with the overall regime [10]. The high medication adherence prevalence rate of 96.8% found in this study could be because more medication adherent patients attended the Centre, being the only tertiary health facility in the locality. Also, there is the possibility that the patients had recall bias about their compliance. This finding is similar to a prevalence rate of 91.44% that was reported in a study conducted among patients with hypertension in the United Kingdom with age range of 40 to 79 years, unlike the reported 43% in a Gambian study [14, 27].

In this study, depression did not affect level of medication adherence. This is contrary to findings in previous studies [29, 33]. Majority of the patients in this study with co-morbid depression had mild depressive episodes which was less likely to affect their level of medication adherence. Also, health talk given by the community health nursing officers before each clinic starts could have influenced the patients level of adherence positively. A study conducted among patients with hypertension in Nigeria reported that poor medication adherence was mainly due to ignorance on need for regular treatment [34]. Prior reports had demonstrated that the presence of depressive symptoms were associated with inadequate blood pressure control and complications of hypertension [6]. Some recent, investigations have reported association between depressive symptoms and low anti-hypertensive medication adherence [22].

## Conclusion

The prevalence of co-morbid depression among patients with hypertension attending the Medical Out-patient Clinic at the Federal Medical Centre, Owo was 22.8%. Mild depressive episode was the commonest form (21.8%) and afflicts nearly three times as many females as the males and the unmarried more than the married. Participants with low level of formal education were more likely to have co-morbid depression than those with higher level of formal education. Also, those with monthly income of < N20,000 were more likely to have depressive episode in comparison with those with monthly income of ≥ N20,000. It was an unexpected finding that the majority of the patients that reported high medication-adherence also had co-morbid depression. Physicians caring for patients with hypertension should routinely screen them for depression which can be easily missed in patients attending hypertensive disease clinic. The detection of co-morbid depression could be facilitated by use of brief questionnaires similar to that used in this study; they should be made available in all consulting rooms because they are easy to administer and score by clinicians. Review of antihypertensive drugs should also be done often to ensure patients are not likely to have depressive illness as a side effect of drugs used. To help patients better understand their health condition and co-operate with their management, regular health talk about co-morbidities and the adverse consequences if undetected, aided with educational video recordings while waiting to see their primary care physicians is strongly recommended. In this study, though unintended, more participants belonged to the age group of ≥ 60 years, reflecting the pattern of patients seen in the centre where this study was carried out. Generalizing the findings here to other centres where younger patients may be more, may not be appropriate. Also, because medication adherence was assessed by self-report, pill counting was not done and experience of side effect was not captured. Ascertainment bias coupled with the problem of recall is noteworthy limitations.

### What is known about this topic

- Prevalence of hypertension in Nigeria;
- Poor adherence to medication among hypertensives;
- Depression has been reported to significantly predict poor medication adherence.

### What this study adds

- The relationship between medication non-adherence and co-morbid depressive disorder in patients with hypertension;
- Importance of routine screening of hypertensive patients for depression;
- Review of antihypertensive drugs should also be done often to ensure patients are not likely to have depressive illness as a side effect of drugs used.

## Competing interests

The authors declare no competing interests.
